# Molecular epidemiology of respiratory viral infections in hospitalized children in Novosibirsk, Russia, 2021–2023

**DOI:** 10.3389/fped.2026.1882419

**Published:** 2026-08-20

**Authors:** Denis Evgenievich Maslov, Ivan Dmitrievich Osipov, Daria Sergeevna Zabelina, Diana Sergeevna Demina, Yulia Eduardovna Tomilova, Sabokhat Bakhromovna Berdieva, Viktoriya Viktorovna Makukha, Tatiana Valerievna Komissarova, Eduard Felixovich Agletdinov, Sergey Viktorovich Netesov

**Affiliations:** 1 Faculty of Natural Sciences, Novosibirsk State University, Novosibirsk, Russia; 2 JSC “Vector-Best”, Novosibirsk, Russia; 3 Faculty of Natural and Agricultural Sciences, Urgench State University, Urgench, Uzbekistan; 4 Children’s Clinical Hospital №3, Novosibirsk, Russia

**Keywords:** acute respiratory infection, adenovirus, molecular epidemiology, pediatric infections, respiratory pathogens

## Abstract

**Background:**

Acute respiratory infections (ARIs) are among the leading global causes of morbidity and mortality in children. While viral agents of ARI are rigorously monitored, other pathogenic organisms and pathobionts — bacteria and fungi native to the host microbiome — remain largely neglected. Adenoviral types also warrant particular attention especially given the widespread use of adenoviral vector vaccines during the COVID-19 pandemic.

**Methods:**

Oro-nasal swabs collected from 1679 children under the age of 18 hospitalized in Novosibirsk, Russia between February 2021 and June 2023, were tested for the presence of nucleic acids by the diagnostic panel targeting 17 viruses, 12 bacteria and one yeast. Adenovirus-positive samples were additionally fine-typed by Sanger sequencing.

**Results:**

Overall, 79.15% of samples were positive for at least one of 17 viral pathogens, with RSV (22.87% positives) and RhV (24.54% positives) dominating the etiological structure. Oro-nasal presence for respiratory pathobionts and opportunistic pathogens was 67.96% and 11.49%, respectively. Generally, viral seasonality was typical for continental climate, with only two notable deviations: MpV absence in spring of 2022 followed by earlier December peaks in the same year; an earlier RSV maximum in October 2021 with extended circulation the next epidemic season. Overall, the epidemiology of the analyzed viruses was consistent with worldwide surveillance trends and largely stable between the two later epidemic seasons. In viral-bacterial association analysis we identified two significantly attracted pairs, in which virus is known to facilitate bacterial adherence to respiratory epithelium. Adenovirus fine-typing provided preliminary evidence that serotypes 1, 2, 3, and 7 are the most frequently detected among the typed samples, while vaccine serotype AdV-5 was identified in only two cases.

**Conclusion:**

Broad target coverage allowed for a comprehensive analysis of epidemiological patterns, highlighting a trend toward stabilization of the etiological structure to pre-pandemic levels, and allowed for prioritization of clinically relevant viral-bacterial associations. Preliminary findings in adenoviral fine-typing are consistent with global trends in serotype prevalence and help address an important gap in regional adenoviral epidemiology.

## 1 Introduction

Acute respiratory infections (ARIs) remain among the leading global causes of morbidity and mortality, placing a substantial burden on public health systems. Each year, an estimated four million deaths are attributed to ARI cases (1, 2), with children under five years of age being the most vulnerable (3). A wide range of pathogens have been described to cause ARIs, including viruses, bacteria, and fungi. While viruses are recognized as the primary causative agents and are subject to rigorous epidemiological surveillance, bacteria and fungi attracted substantially less attention. These organisms represent a diverse and clinically significant group, encompassing primary respiratory pathogens, such as *Bordetella spp.* and atypical bacteria; opportunistic pathogens such as *Klebsiella pneumoniae* and *Pseudomonas aeruginosa*; but dominated by conditionally pathogenic bacteria, common to the host microbiome (4). Interpreting the detection of this latter group presents a challenge as a positive signal often represents asymptomatic carriage rather than primary cause of infection. Nevertheless, the presence of these organisms is clinically relevant, as their carriage serves as a reservoir for potential superinfection and is associated with increased severity of the viral disease course (5).

Since the surge of severe acute respiratory syndrome coronavirus 2 (SARS-CoV-2) that caused the COVID-19 pandemic the epidemiological landscape of ARI changed. Circulation of SARS-CoV-2, combined with widespread introduction of non-pharmaceutical interventions, such as social distancing and mask use, has substantially modified the incidence and seasonality of other respiratory viruses (6, 7). Shifts in the etiological landscape of other ARIs may also be driven by mechanisms such as viral interference with SARS-CoV-2 or the effects of antiviral vaccines (8, 9). Moreover, the epidemiology of adenoviruses has been specifically impacted by mass immunization employing adenoviral vectors, such as a widely used Ad5/Ad26-based Sputnik V (10).

In the present study, we described the epidemiological patterns of ARIs among pediatric in-patients hospitalized in Novosibirsk, Russia during the period from 8 February 2021 to 27 June 2023, encompassing the rise and decline of COVID-19 pandemic. To the best of our knowledge, we employed the broadest analytical panel used in Russia for detection of primary and conditional ARI pathogens, capturing 17 viral, 12 bacterial, and one fungal target. Additionally, we performed fine-typing of adenoviruses to monitor circulation patterns of specific serotypes, particularly those utilized in vector-based vaccines. These findings may contribute to a more comprehensive understanding of respiratory pathogen dynamics in pediatric populations during and following major public health disruptions.

## 2 Materials and methods

### 2.1 Ethics statement

All research procedures were performed in accordance with the 1964 Declaration of Helsinki and its subsequent amendments and national standards. The study was approved by the Local Ethics Committee of Novosibirsk State University, Novosibirsk, Russia (excerpt from Ethics Committee protocol no. 1, dated September 12, 2021). Written informed consent was obtained from the legal representatives of all patients. As specified in the research proposal and the approved study protocol, the study also included analysis of residual nucleic acids extracted from respiratory specimens collected during routine clinical care. No additional sampling or study-specific procedures were performed.

### 2.2 Clinical data and sample collection

All respiratory samples were obtained from children under the age of 18 admitted to Novosibirsk Children's Municipal Clinical Hospital №3 between 8 February 2021 and 27 June 2023. Patients were enrolled in the study if they presented with at least one systemic symptom (fever, headache, myalgia) and one respiratory symptom (cough, rhinorrhea, nasal congestion, sore throat, shortness of breath, lung auscultation abnormalities, or chest pain). Patients lacking signed informed consent made by their legal representatives (or with one retracted) were excluded.

The diagnosis of ARI was established through a comprehensive evaluation of patient complaints, clinical features, anamnestic history (an acute onset, exposure to ARI cases within the family or childcare/school environment, occurrence during the autumn–winter season), and results from laboratory tests and instrumental studies. Laboratory testing was performed on all patients, regardless of disease severity, and included a complete blood count with differential, urinalysis, and blood biochemistry. Instrumental investigations—otoscopy, radiography (plain x-ray), and computed tomography—were utilized when clinically indicated. For the downstream analysis of clinical features, we divided patients into 3 groups according to their ARI clinical profile: (a) upper respiratory tract infection (URT), (b) lower respiratory tract infection (LRT), and (c) pneumonia subset of LRT (Lung). The URT group included cases diagnosed by physicians as tonsillitis, rhinopharyngitis, laryngitis, croup and any cases of unspecified ARI. The LRT group included bronchiolitis, bronchitis, obstructive bronchitis and laryngotracheitis, with cases of pneumonia categorized separately.

Paired nasal and oral swabs were taken from each enrolled patient by a hospital nurse no later than three days after admission. Samples were placed in tubes with guanidine-based Transport solution M (JSC Vector-Best, Novosibirsk, Russia), and were stored at 4 °C for no more than 48 h before nucleic acid extraction.

### 2.3 PCR detection

Nucleic acids were extracted from oro-nasal swabs using the RealBest Sorbitus kit (JSC Vector-Best, Novosibirsk, Russia) in accordance with the manufacturer's instructions. Briefly, oral and nasal swabs were collected in 2 mL of transport medium, 100 μL were used for total nucleic acid extraction. Sample quality was assessed using the RealBest Sample Validation kit (#D-8888, JSC Vector-Best, Novosibirsk, Russia) which targets the human *HMBS* gene as an internal control. Samples with an *HMBS* Ct value greater than 33 were considered inadequate and excluded from further analysis in accordance with the manufacturer's instructions. Detection of respiratory viruses, namely, parainfluenza virus types 1–4 (PIV1–4), respiratory syncytial virus (RSV), human metapneumovirus (MpV), seasonal coronaviruses OC43, 229E, NL63, and HKU1, (CoV-OC43, CoV-229E, CoV-NL63, and CoV-HKU1), influenza A and B viruses (IFV-A and IFV-B), rhinovirus (RhV), Human Mastadenovirus species B, C, and E^1^ (AdV), bocavirus (BoV), non-rhinovirus enteroviruses (EntV), and SARS-CoV2, was performed by RT-PCR using RealBest-ARVI RNA hPIV1/3 (#D-5563), RealBest-ARVI RNA hRSV/hPIV4 (#D-5567), RealBest-ARVI RNA hMpV/hPIV2 (#D-5560), RealBest-ARVI RNA hCoV OC43/HKU1 (#D-5565), RealBest-ARVI RNA hCoV 229E/NL63 (#D-5566), RealBest-ARVI RNA Influenza virus A/B (#D-5564), RealBest-ARVI RNA hRV (#D-5561), RealBest-ARVI DNA hAdV/hBoV (#D-5562), RealBest RNA Enterovirus (#D-1695) and RealBest RNA SARS-CoV-2 (#D-5580) kits (JSC Vector-Best, Novosibirsk, Russia) in accordance with the manufacturer's instructions. Detections of bacteria of the *Bordetella* genus, *Chlamydia* (*Chlamydophila*) *pneumoniae*, *Mycoplasma pneumoniae,* and *Legionella pneumophila* was carried out by RealBest DNA Bordetella species/Bordetella pertussis/Bordetella bronchiseptica (#D-5586), RealBest DNA Chlamydophila pneumoniae (#D-5594), RealBest DNA Mycoplasma pneumoniae (#D-5596) and RealBest DNA Legionella pneumophila (#D-5598) kits (JSC Vector-Best, Novosibirsk, Russia). Additionally, we evaluated the oro-nasal prevalence of bacteria and fungi with a potential role in ARI pathogenesis namely *Haemophilus influenzae*, *Klebsiella pneumoniae*, *Neisseria meningitidis*, *Pseudomonas aeruginosa*, *Streptococcus pneumoniae*, *Staphylococcus aureus*, and the yeast *Candida albicans*, by the using RealBest DNA Haemophilus influenzae (#D-5592), RealBest DNA Klebsiella pneumoniae/Pseudomonas aeruginosa (#D-5605), RealBest DNA Neisseria meningitidis (#D-5584), RealBest DNA Streptococcus pneumoniae (#D-5590), RealBest DNA Staphylococcus aureus/mecA/lukS-PV (#D-5603) and RealBest DNA Candida albicans/Fungi (#D-0448) kits (JSC Vector-Best, Novosibirsk, Russia), correspondingly. *Moraxella catarrhalis* and *Streptococcus pyogenes* were detected with a research use only kit (not marked, JSC Vector-Best, Novosibirsk, Russia). Detection of the mecA gene defining bacterial methicillin resistance was carried out by the RealBest DNA Staphylococcus aureus/mecA/lukS-PV (#D-5603) kit (JSC Vector-Best, Novosibirsk, Russia). Positive and negative controls were included in each run.

For clarity of the downstream analysis, the aforementioned pathogens were classified into three groups based on their typical colonization patterns and pathogenic behavior in the respiratory tract: (a) primary respiratory pathogens group, which included all viral targets and *Bordetella spp.*, *C. pneumoniae*, *M. pneumoniae*, and *L. pneumophila*; (b) opportunistic pathogens group, which included environmental opportunists *K. pneumoniae* and *P. aeruginosa*; (c) respiratory tract pathobionts, which included habitual upper-respiratory tract resident bacteria *H. influenzae*, *M. catarrhalis*, *S. pneumoniae*, *S. aureus*, *N. meningitidis*, *S. pyogenes*^2^*,* and the commensal yeast *C. albicans*.

As microorganisms in the latter group may colonize the upper respiratory tract, their PCR detection was interpreted as evidence of microbial presence rather than clinically confirmed infection, since a positive signal may reflect either asymptomatic carriage or active infection.

### 2.4 Adenovirus fine-typing

An in-house system was used for Adenovirus fine-typing. The hexon gene fragment was amplified and Sanger-sequenced using the following primers (nucleotide positions are relative to the AdV-5 hexon sequence, GenBank accession no. AB330086.1): amplification forward 5′-CAGGAYGCYTCGGAGTACCT-3′ (52–71 nt), amplification reverse 5′-GCIGTGTTGTGIGCCAT-3′ (1,864–1,880 nt) and sequencing primer 5′-GTGCAGTTYGCCCGTGCAAC-3′ (85–104 nt). PCR products were extracted using AMPure XP Beads (Beckman Coulter, USA) and put for sequencing using NovaDye Terminator Cycle Sequencing Kit v3.1 (GeneQuest, Moscow, Russia) and 3,500 Series Genetic Analyzer (Applied Biosystems, USA).

All sequence were deposited into the VGARus genomic database under identifiers novg000053–novg000099 and GenBank under accessions PZ797416–PZ797462, see Supplementary Table 1 for description.

Raw sequencing data were processed by Sequencing Analysis Software v6.0 (Applied Biosystems, USA) to generate nucleotide sequences. These sequences were combined with a set of reference hexon gene sequences (ncbi accessions: C1—AF534906.1, C2—MF044052.1, C5—KX868466.2, C6—LC068720.1, B7—MH910669.1, B16—JN860680.1, B11—AF532578.1, B14—MF062484.1, B55—MK123981.1, B3—KX384958.1, B21—MF502426.1, B35—AY271307.1, E4—AP014853.1, A12—NC_001460.1, D8—MH634393.1), pretrimmed to only contain the extended target region 100–1,100, and aligned using mafft v7.490 (13) with the L-NSI-i (–localpar) strategy. Multiple sequence alignment was trimmed by trimAl v1.2 (option -automated1) (14), and the phylogeny was constructed by iqtree v2.2.0.5 (15), using the ModelFinder module (16) to identify TPM2u + F + I + G4 model as optimal, with 100 nonparametric bootstrap replicates, rooted with the sequence from serotype 12 (species A). The phylogeny was visualized and annotated using the Interactive Tree Of Life (iTOL) v7 web server (17). Final phylogenetic assignment was determined based on top BLAST hit against NR database and the sequence's placement within a specific serotype clade.

### 2.5 Data analysis

Data were summarized as prevalences (percentages) with 95% confidence interval (95% CI) calculated using the Clopper-Pearson method. A significance level of *α* = 0.05 was used for hypothesis testing, with appropriate adjustments applied to control for multiple testing.

Pairwise comparisons of prevalence between epidemic and interepidemic intervals were performed using Fisher's exact test. Differences between epidemic seasons were analyzed by logistic regression with interval as the predictor. Prevalences across different age groups were analyzed by logistic regression, with age group as the predictor. Statistical significance was first assessed using an omnibus Likelihood Ratio Test, if significant, pairwise comparisons were subsequently performed using Tukey's range test. Associations between prevalence and infection site were analyzed using a logistic regression with location treated as an ordered factor. The omnibus test was considered passed if either the linear or quadratic coefficients were significant, and followed by Tukey's range test for *post-hoc* analysis. For the analyses described above, the Family-Wise Error Rate (FWER) was controlled using the Bonferroni adjustment. Because patient age is associated with both the site of the infection and the prevalence of several pathogens, association for pathogens whose prevalence was significantly associated with infection site were re-analyzed using logistic regression models additionally adjusted for age group and sampling interval. Significance of the linear and quadratic coefficients was assessed using the same Bonferroni adjustment (using the same family size) as in the primary analysis. A site association was considered robust if the trend term significant in the primary analysis (linear or quadratic) retained both significance and direction after adjustment.

Pairwise co-detections were analyzed using Fisher's exact test. The analysis was restricted to pairs of pathogens with a co-detection count of at least ten. To account for the increased number of tests, we controlled for the False Discovery Rate (FDR) using the Benjamini-Hochberg procedure. To assess whether the significant pairwise co-detections were confounded by age and sampling period, each significant pair was re-analyzed by logistic regression in both directions using age group and sampling periods as covariates: pathogen A was modeled as a function of pathogen B and covariates, and A and B exchanged in the symmetric model. Age- and interval-adjusted odds ratios (ORs) were obtained. An association was considered robust if it retained both direction and statistical significance (Wald's test for the other pathogen) in both modeling directions. FDR was controlled across all direction-specific tests using the Benjamini-Hochberg procedure.

Association between severity and confirmed co-associated pairs was tested by logistic regression, using infection site as a proxy for severity (LRT or Lung were considered severe; otherwise, mild). The model included effects for individual pathogens and their interaction. We specifically evaluated the significance of the interaction coefficient, applying a Bonferroni adjustment.

## 3 Results

### 3.1 Virus and pathobiont detection

**Figure 1:**
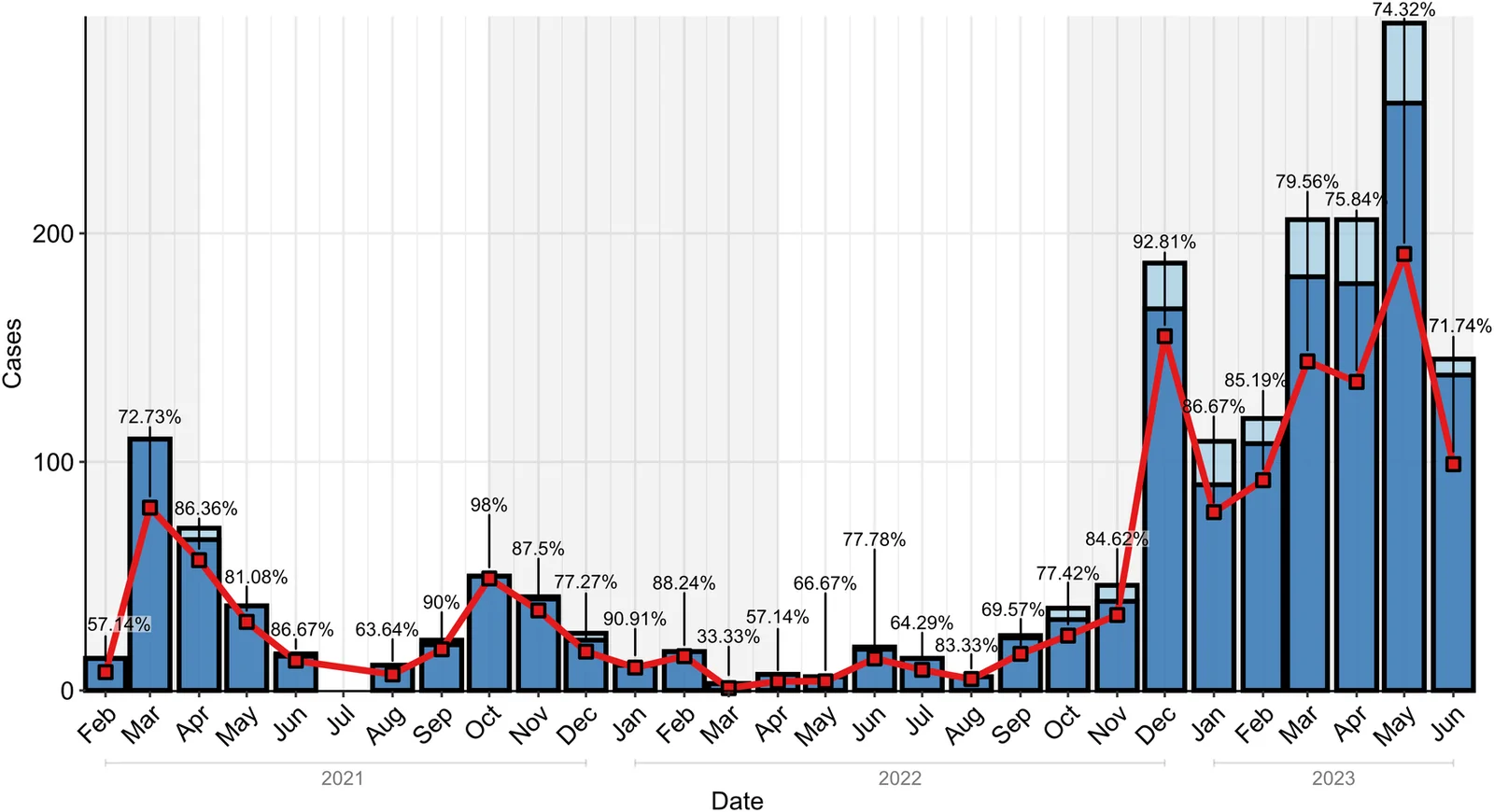
Monthly detection positivity rate. Stacked bar plots represent the number of samples collected each month. Light blue bars indicate the total number of collected samples (including those excluded from the analysis), dark blue bars indicate the number of samples included in the analysis. The red markers represent the number of positive samples with the corresponding positivity rate (%) indicated for each month.

A total of 1,850 children under eighteen years of age were included in the study from February 2021 to June 2023, a span that traversed the rise and decline of the COVID-19 pandemic. The study window included three epidemic and two interepidemic seasons. For analytical coherence, the dataset was divided into five intervals: February–April 2021 (hereinafter, E21), representing the second half of the 2020–2021 epidemic season; May–September 2021 (I21), interepidemic; October 2021–April 2022 (E22); May–September 2022 (I22); and October 2022–June 2023 (E23), the final epidemic season, extended by May and June, overlapping with the WHO's declaration of the end of the COVID-19 global health emergency in May, 2023 (18). One hundred seventy-one samples were excluded from downstream analysis due to insufficient human DNA concentration, indicative of poor sampling quality, leaving 1,679 samples for the evaluation. The temporal distribution across the specified intervals was uneven, with 70% of observations being sampled in E23, 20% during E21 and E22 combined, and the two inter-epidemic seasons accounted for the remainder (see Figure 1 for monthly numbers of tested patients). Age distribution was stable across intervals; in each, children under two years comprised roughly one-half to two-thirds of observations (Supplementary Table 2). The overall median age was 12 months (IQR: 5 months – 3 years). Males represented 58.82% of the cohort, without substantial differences across different age groups or intervals.

Of the 1,679 samples included in the analysis 1,347 (80.22%, 95% CI [78.25, 82.06]) were positive for at least one primary respiratory pathogen (Table 1, Supplementary Table 2). Detection rates fluctuated between 71.64% and 87.33% across intervals, showing no overall temporal trend, except for a single significant difference between E22 and I22 (OR=2.73, $p_{adj}=0.0476$). Positivity declined with age: children in the age bracket of 7–17 years were 1.3-fold less likely to test positive than those under seven $\left(\;p_{adj}=5.71\times 10^{-6}\right)$. Across the study window, picornaviruses were dominant, identified in 27.69% of samples (n=465, 95% CI [25.56%, 29.90%]) with minimal overlap between RhV and EntV. Pneumoviruses—RSV and MpV,—followed, detected in 22.87% (n=384, 95% CI [20.88, 24.95]) and 6.13% (n=103, 95% CI [5.03, 7.39]) of samples, respectively. DNA of BoV and AdV was identified in 13.22% (n=222, 95% CI [11.64, 14.94]) and 9.53% (n=160, 95% CI [8.17, 11.03]) samples. Parainfluenza viruses (PIV) were detected in 10.06% (n=169, 95% CI [8.67, 11.60]) of participants, predominantly type 3 (69.8 % of those infections). The marginal prevalence of influenza viruses was 8.75% (n=147, 95% CI [7.45, 10.21]), with IFV-A being three-fold more common than IFV-B. Seasonal coronaviruses accounted for 5.54% (n=93, 95% CI [4.49, 6.74]) cases, while SARS-CoV-2 was detected in 7.09% (n=119, 95% CI [5.91, 8.42]) of individuals. Bacterial pathogens were rare, identified in only 18 participants: 13 cases of *Bordetella spp.* infections and five cases of other atypical bacteria, with zero cases for *L. pneumophila* infection*.* None of these 18 bacterial cases had a concurrent viral detection, so 1,329 (79.15%, 95% CI [77.13, 81.07]) were positive for at least one viral pathogen.

**Table 1:** Detection counts and marginal prevalences of primary respiratory pathogens, opportunistic pathogens, and respiratory pathobionts by sampling interval (E21, I21, E22, I22, E23) and overall, among 1679 oro-nasal samples from hospitalized children, Novosibirsk, Russia, 2021–2023.

Pathogen	Count (%)					
	E21 (*N* = 190)	I21 (*N* = 83)	E22 (*N* = 150)	I22 (*N* = 67)	E23 (*N* = 1,189)	Total (*N* = 1,679)
**Primary pathogens**	145 (76.32)	68 (81.93)	131 (87.33)	48 (71.64)	955 (80.32)	1,347 (80.23)
IFV-A	3 (1.58)	0	19 (12.67)	0	89 (7.49)	111 (6.61)
IFV-B	0	0	0	1 (1.49)	35 (2.94)	36 (2.14)
PIV-1	0	0	0	4 (5.97)	11 (0.93)	15 (0.89)
PIV-2	4 (2.11)	0	2 (1.33)	0	19 (1.60)	25 (1.49)
PIV-3	4 (2.11)	12 (14.46)	2 (1.33)	2 (2.99)	98 (8.24)	118 (7.03)
PIV-4	5 (2.63)	2 (2.41)	0	2 (2.99)	3 (0.25)	12 (0.71)
MpV	48 (25.26)	0	1 (0.67)	0	54 (4.54)	103 (6.13)
RSV	2 (1.05)	16 (19.28)	73 (48.67)	1 (1.49)	292 (24.56)	384 (22.87)
AdV	5 (2.63)	4 (4.82)	19 (12.67)	5 (7.46)	127 (10.68)	160 (9.53)
BoV	41 (21.58)	32 (38.55)	23 (15.33)	7 (10.45)	119 (10.01)	222 (13.22)
CoV-229E	4 (2.11)	0	3 (2.00)	1 (1.49)	6 (0.50)	14 (0.83)
CoV-NL63	16 (8.42)	1 (1.20)	1 (0.67)	0	22 (1.85)	40 (2.38)
CoV-OC43	17 (8.95)	1 (1.20)	0	0	24 (2.02)	42 (2.50)
CoV-HKU1	0	0	0	0	0	0
SARS-CoV-2	4 (2.11)	2 (2.41)	15 (10)	6 (8.96)	92 (7.74)	119 (7.09)
RhV	39 (20.53)	23 (27.71)	23 (15.33)	29 (43.28)	298 (25.06)	412 (24.54)
EntV	1 (0.53)	2 (2.41)	5 (3.33)	6 (8.96)	39 (3.28)	53 (3.16)
*Bordetella spp.*	0	0	0	1 (1.49)	12 (1.01)	13 (0.77)
*C. pneumoniae*	0	0	0	1 (1.49)	2 (0.17)	3 (0.18)
*M. pneumoniae*	0	0	0	0	2 (0.17)	2 (0.12)
*L. pneumophila*	0	0	0	0	0	0
**Opportunistic pathogens**	21 (11.05)	6 (7.23)	16 (10.67)	13 (19.40)	137 (11.52)	193 (11.49)
*K. pneumoniae*	12 (6.32)	4 (4.82)	6 (4.00)	5 (7.46)	99 (8.33)	126 (7.50)
*P. aeruginosa*	10 (5.26)	3 (3.61)	10 (6.67)	9 (13.43)	52 (4.37)	84 (5.00)
**Respiratory pathobionts**	128 (67.37)	45 (54.22)	92 (61.33)	48 (71.64)	828 (69.64)	1,141 (67.96)
*H. influenzae*	30 (15.79)	11 (13.25)	28 (18.67)	23 (34.33)	292 (24.56)	384 (22.87)
*M. catarrhalis*	58 (30.53)	13 (15.66)	55 (36.67)	25 (37.31)	316 (26.58)	467 (27.81)
*S. pneumoniae*	72 (37.89)	23 (27.71)	38 (25.33)	25 (37.31)	376 (31.62)	534 (31.80)
*S. aureus*	27 (14.21)	7 (8.43)	19 (12.67)	14 (20.9)	173 (14.55)	240 (14.29)
*N. meningitidis*	2 (1.05)	1 (1.20)	0	0	13 (1.09)	16 (0.95)
*S. pyogenes*	7 (3.68)	4 (5.82)	5 (3.33)	4 (5.97)	81 (6.81)	101 (6.02)
*C. albicans*	26 (13.68)	9 (10.84)	23 (15.33)	9 (13.43)	161 (13.54)	228 (13.58)
mecA	42 (22.11)	16 (19.28)	19 (12.67)	9 (13.43)	246 (20.69)	332 (19.77)

IFV(-A/-B), influenza virus A/B; PIV(-1/-2/-3/-4), human parainfluenza virus 1/2/3/4; MpV, human metapneumovirus; RSV, human respiratory syncytial virus; AdV, Human mastadenovirus of species C, B, or E; BoV, human bocavirus; CoV(-229E/-NL63/OC43/HKU1), human coronavirus 229E/NL63/OC43/HKU1; SARS-CoV-2, severe acute respiratory syndrome coronavirus 2; RhV, human rhinovirus; EntV, non-rhinovirus enteroviruses; mecA, gene for methicillin resistance; E21, February–April 2021; I21, May–September 2021; E22, October 2021–April 2022; I22, May–September 2022; E23, October 2022–June 2023.

Respiratory pathobionts were detected in 67.96% of participants (n=1141, 95% CI [65.66, 70.18], Table 1, Supplementary Table 2). The marginal prevalence of individual species was 22.87% for *H. influenzae* (n=384, 95% CI [20.88, 24.95]), 27.81% for *M. catarrhalis* (n=467, 95% CI [25.68, 30.02]), 31.80% for *S. pneumoniae* (n=534, 95% CI [29.58, 34.09]), 14.29% for *S. aureus* (n=240, 95% CI [12.65, 16.06]), 6.02% for *S. pyogenes* (n=101, 95% CI [4.93, 7.26]), 13.58% for the yeast *C. albicans* (n=228, 95% CI [11.97, 15.31]), and only 0.95% for *N. meningitidis* (n=16, 95% CI [0.55, 1.54]). DNA of opportunists *K. pneumoniae* and *P. aeruginosa* was identified in 193 samples (11.49%, 95% CI [10.01, 13.12], Table 1, Supplementary Table 2), with marginal prevalences of 7.50% (n=126, 95% CI [6.29, 8.88]) and 5.00% (n=84, 95% CI [4.01, 6.16]) for each of them separately.

### 3.2 Primary respiratory pathogen temporal distribution

**Figure 2:**
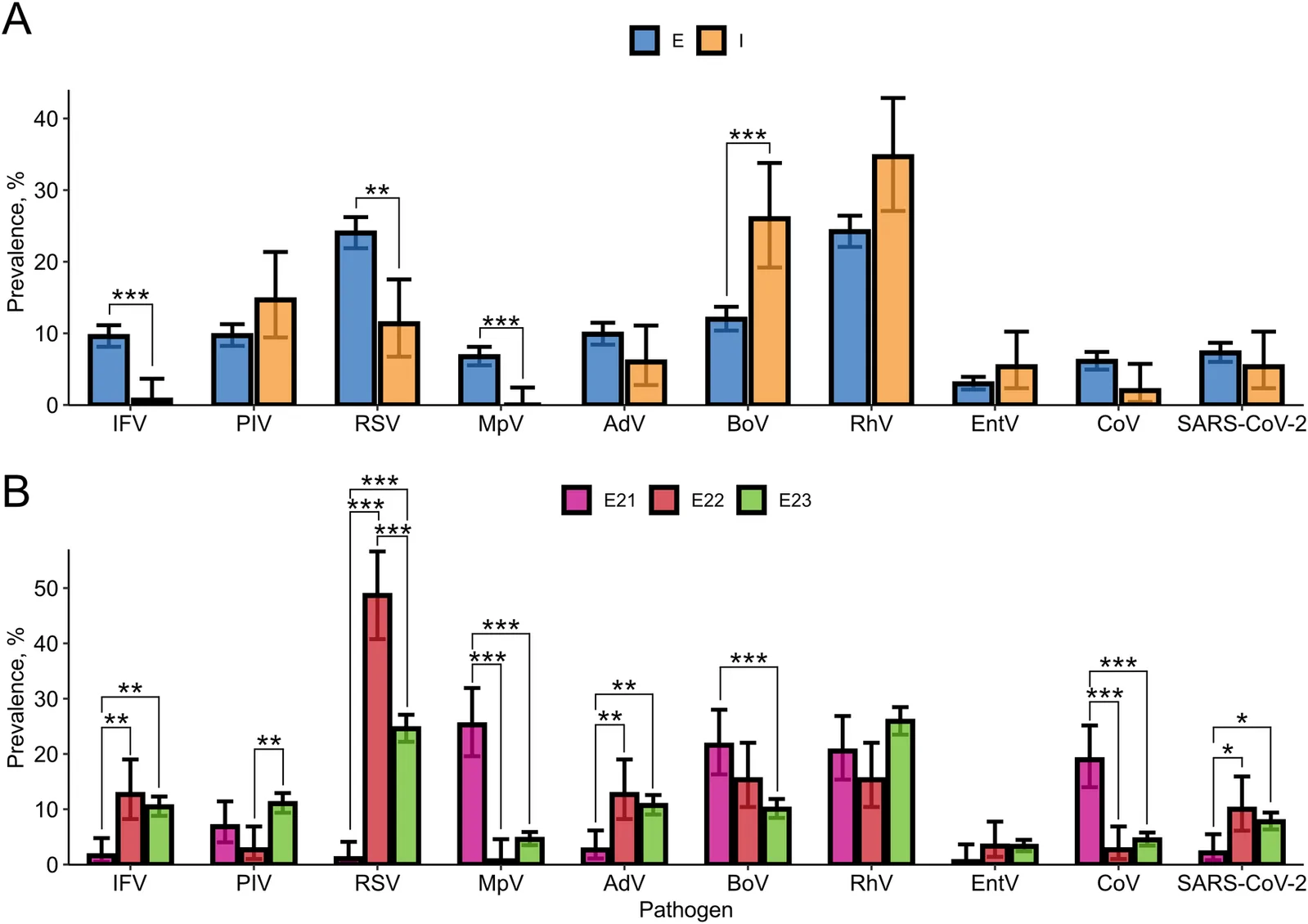
Primary respiratory pathogens' marginal prevalences across different time intervals. **(A)** Prevalences compared between three epidemic (E21, E22, E23) and two interepidemic seasons (I21, I22). Data are presented as prevalences with 95% CI calculated by the Clopper-Pearson method. Comparisons between groups were performed using Fisher's exact test, with *p*-values adjusted by Bonferroni correction. **(B)** Prevalences compared between three epidemic season (E21, E22, E23). Data are presented as prevalences with 95% CI calculated by the logit-transformation method. Overall group differences were evaluated using the likelihood ratio test; *p*-values were Bonferroni-adjusted for multiple testing. If the likelihood ratio test was significant, prevalence pairwise comparisons were performed by Tukey's range test. Significance levels: *p_adj_ < 0.05, ** *p_adj_* < 0.01, ***p_adj_ < 0.001. IFV, influenza virus; PIV, human parainfluenza virus 1/2/3/4; MpV, human metapneumovirus; RSV, human respiratory syncytial virus; AdV, human mastadenovirus of species C, B, or E; BoV, human bocavirus; CoV, human seasonal coronavirus; SARS-CoV-2, severe acute respiratory syndrome coronavirus 2; RhV, human rhinovirus; EntV, non-rhinovirus enteroviruses; E21, February–April 2021; I21, May–September 2021; E22, October 2021–April 2022; I22, May–September 2022; E23, October 2022–June 2023.

**Figure 3:**
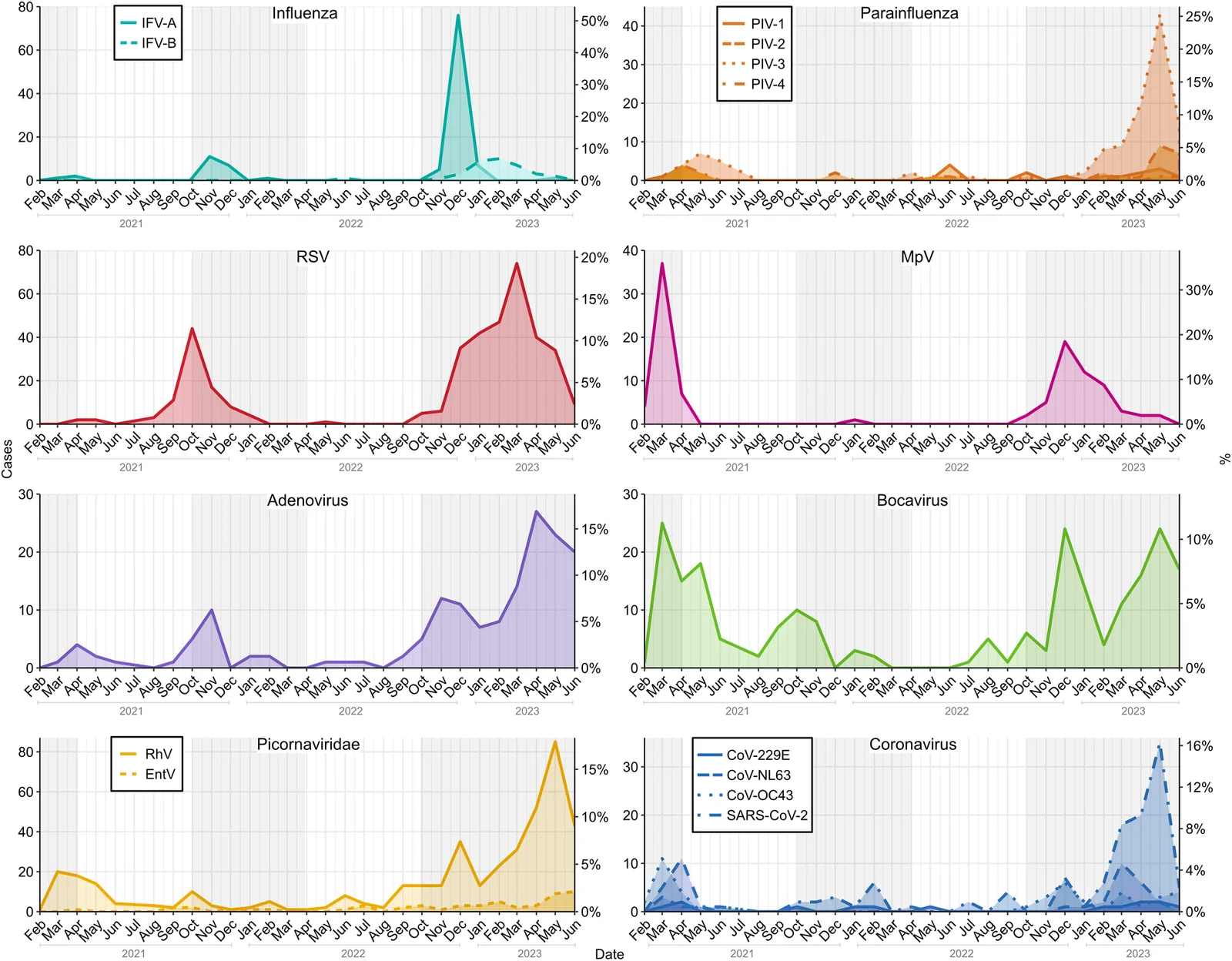
Seasonality of respiratory viruses. The left *Y*-axis denotes the number of positive cases in a given time point; the right *Y*-axis gives the percentage relative to total positive cases for the pathogen. IFV(-A/-B), influenza virus A/B; PIV(-1/-2/-3/-4), human parainfluenza virus 1/2/3/4; MpV, human metapneumovirus; RSV, human respiratory syncytial virus; AdV, human mastadenovirus of species C, B, or E; BoV, human bocavirus; CoV(-229E/-NL63/OC43/HKU1): human coronavirus 229E/NL63/OC43/HKU1; SARS-CoV-2, severe acute respiratory syndrome coronavirus 2; RhV, Human rhinovirus; EntV, non-rhinovirus enteroviruses.

We compared the prevalence and temporal distribution of primary respiratory pathogens. Due to low overall prevalence, bacterial cases were excluded, and for the same reason, seasonal coronaviruses (CoV), parainfluenza virus types (PIV), and influenza virus subtypes (IFV) were analyzed jointly within their respective groups. As anticipated, IFV, RSV, and MpV were significantly more prevalent during winter epidemic seasons (Figure 2A), each followed the continental pattern of winter ascent and spring decline, with minor deviations (Figure 3). Before the COVID-19 pandemic, IFV incidence in Russia typically peaked in December (weeks 49–51); similar maxima were observed in E22 and E23, located in November and December, respectively. The E21 interval began after the supposed maxima, and thus the study captured only its potential decline. RSV showed an unusual temporal pattern: a premature peak in October of 2021 (mid I21-E22), followed by a more conventional peak that spanned the whole winter and included an additional maximum in March. MpV retained its March peak in E21, was almost absent in the winter-spring of E22, and shifted towards December in E23. BoV, on the other hand, demonstrated a significantly higher prevalence in interepidemic intervals (Figure 2A), while retaining its characteristic winter peak. AdV was detected through the year with a modest increase in its prevalence in epidemic seasons. PIVs were relatively rare in the data and showed no significant differences between epidemic and non-epidemic intervals (Figure 2A), although PIV-3 maintained its spring-summer peak. Seasonal coronaviruses were similarly poorly represented in the data and exhibited only a nominal winter increase (nominal *p* = 0.0454), with most cases falling into the winter-spring of E21. SARS-CoV-2 was relatively rare, detected mostly during epidemic seasons, without statistically significant seasonal variation. In the spring of the final interval (E23), several year-round or inter-epidemic pathogens—PIV-3, RhV, AdV and BoV—showed a renewed increase, as did SARS-CoV-2, with a third of its detections falling to March–May 2023.

We next compared the marginal prevalence of each virus across the three epidemic seasons (Figure 2B, Supplementary Table 2). Eight of ten differed significantly across seasons (Likelihood Ratio Test). The difference was predominantly driven by the E21 interval, which spanned only February–April 2021, the tail of a single season, behaving therefore as a systematic outlier. For the classic winter pathogens RSV and IFV, prevalence in E21 was significantly lower than in either subsequent season (1.05% in E21, 48.67% in E22, and 24.56% in E23 for RSV, 1.57%, 12.67%, and 10.43% for IFV), consistent with the E21 interval having missed their early-winter peak. The converse was the case for MpV and seasonal coronaviruses, whose spring maxima fell within the E21 window: both were significantly elevated in E21 and lowered thereafter with no significant difference between E22 and E23 (25.26% in E21, a single case in E22, 4.54% in E23 for MpV, 18.94%, 2.67%, and 4.46% for coronaviruses). AdV and SARS-CoV-2 were also significantly less prevalent in E21 than in E22 and E23 (2.63% in E21, 12.67% in E22, and 10.68% in E23 for AdV, 2.11%, 10.00%, and 7.74% for SARS-CoV-2), although to a lesser extent. Of the eight viruses with a significant omnibus test, only PIV and RSV changed significantly between E22 and E23: PIV rose fourfold between E22 and E23, from 2.67% to 11.02%, driven almost entirely by PIV-3, while being stable between E21 and E22; RSV declined from an anomalously high prevalence in E22, to common pediatric prevalence, so that E23 most likely represents a regression toward the norm. BoV showed a monotonic decline through E21–E23, with E21 prevalence being twice as high as E23 (21.58% and 10.01%, respectively). RhV and EntV were the only viruses that did not differ significantly across epidemic seasons.

### 3.3 Primary respiratory pathogen distribution across different age groups

**Figure 4:**
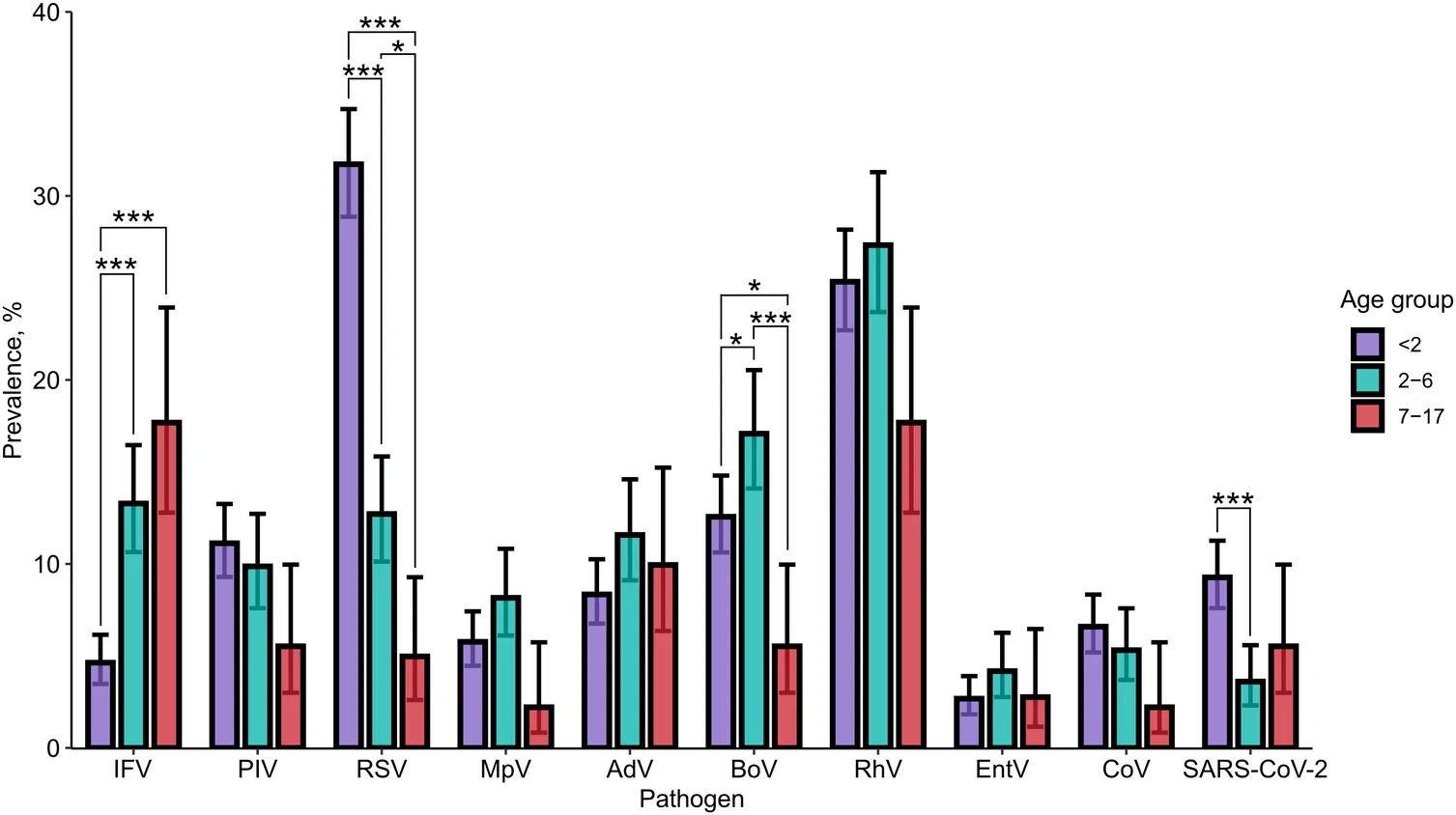
Primary respiratory pathogens' marginal prevalences across different age groups. Data are presented as prevalences with 95% CI calculated using the logit-transformation method. Prevalences were analyzed by a logistic model. Overall group differences were evaluated using the likelihood ratio test; *p*-values were Bonferroni-adjusted for multiple testing. If the likelihood ratio test was significant, prevalence pairwise comparisons were performed by Tukey's range test. Significance levels: *p_adj_ < 0.05, ** *p_adj_* < 0.01, ***p_adj_ < 0.001. IFV, influenza virus; PIV, human parainfluenza virus 1/2/3/4; MpV, human metapneumovirus; RSV, human respiratory syncytial virus; AdV, human mastadenovirus of species C, B, or E; BoV, human bocavirus; CoV, human seasonal coronavirus; SARS-CoV-2, severe acute respiratory syndrome coronavirus 2; RhV, human rhinovirus; EntV, non-rhinovirus enteroviruses.

Comparison across age groups had shown significant differences in the prevalence of BoV, IFV, RSV, and SARS-CoV-2 (Figure 4, Supplementary Table 2). RSV demonstrated a sharp decrease in its prevalence with age, declining from 31.72%, the highest marginal prevalence observed across all viruses and age groups, in children under two years of age to 4.97% in school-aged children (7–17 years). IFV demonstrated an opposite trend, rising from 4.63% in children under two to 13.28% in preschoolers (2–6 years) and to 17.68% in the age bracket of 7–17 years. SARS-CoV-2 was more common in children under two years of age, than in preschoolers (9.26% vs. 3.61%, $p_{adj}=\;0.0003$); the other two pairwise comparisons were not significant. Unlike viruses above, age-specific prevalence pattern of BoV was not monotonic, with maximum prevalence of 17.08% achieved in the 2–6 age bracket, significantly higher than the 7–17 bracket (5.52%, $p_{adj}\;=\;0.0008$) and the under two group (12.56%, $p_{adj}=\;0.0450$); the difference between the latter two groups was also significant $\left(\;p_{adj}\;=\;0.0220\right)$.

### 3.4 Distribution of viral and bacterial detections across upper- and lower-tract infections

We analyzed the association between pathogen prevalence and the clinical site of infection. Participants had been classified into three categories based on diagnosis: (a) upper respiratory tract infection (URT, n=1,167), (b) lower respiratory tract infection (LRT, n=380), and (c) pneumonia subset of LRT (Lung, n=132, subtracted from LRT). Infection site is associated with the severity of ARI, and often implies distinct etiological profiles (19, 20), although these anatomical categories should not be equated with a validated severity grade. We, therefore, tested for an ordered trend in pathogen prevalence across respiratory tract (URT, LRT, Lung, Supplementary Table 3). Pathogens with zero counts in any category had been excluded to avoid the “complete separation” scenario in the model, or in case of PIV, IFV, and CoV, were aggregated by family. *N. meningitidis* had been excluded due to its extremely low prevalence (see Table 1, Supplementary Table 2, 3). Among the excluded pathogens, *Bordetella spp.* was detected in eleven URT cases and two pneumonic cases; *C. pneumoniae* appeared in three URT cases only; *M. pneumoniae* in one URT and one pneumonic case; *N. meningitidis* in six URT, three LRT, and seven pneumonic cases.

**Figure 5:**
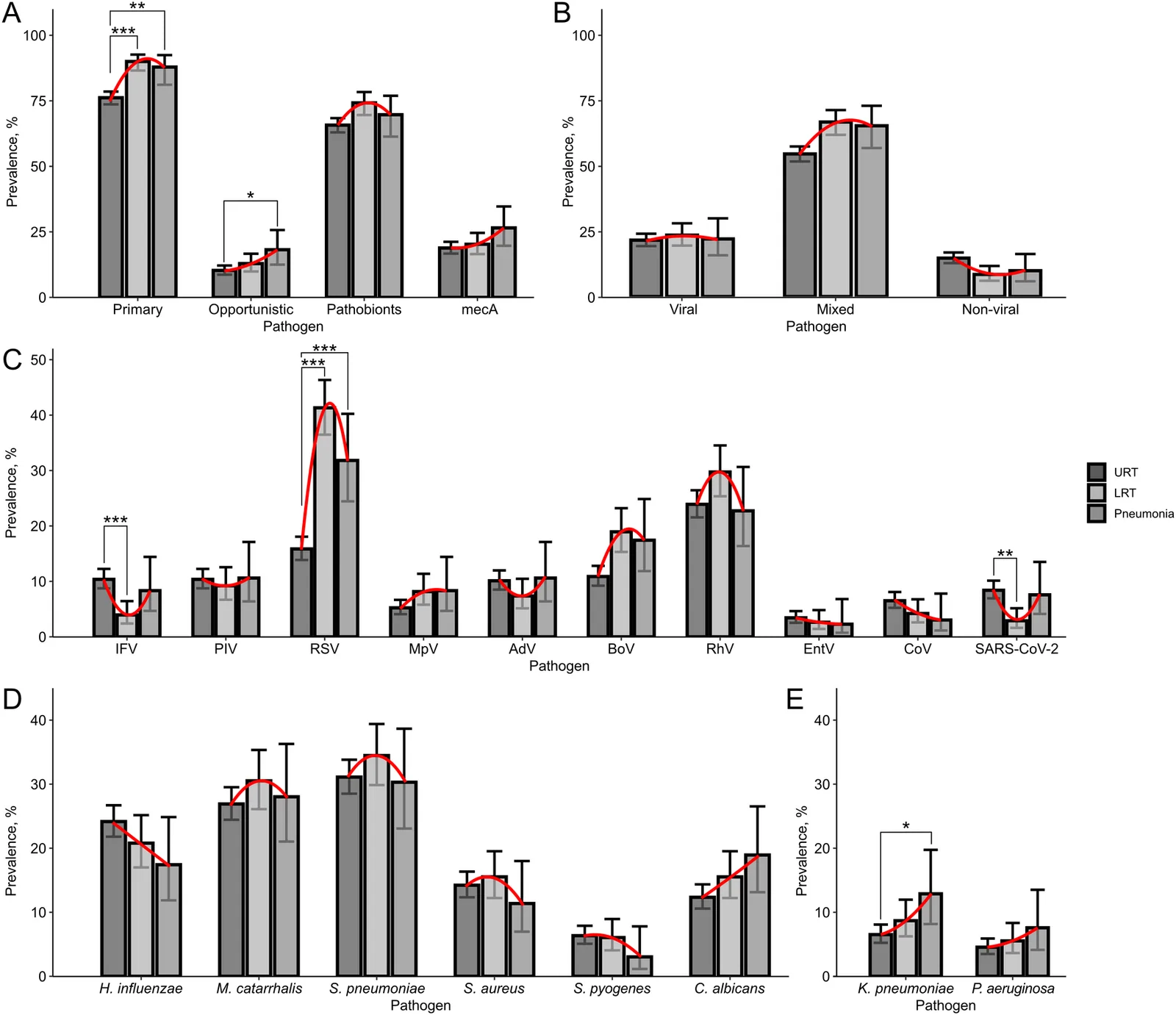
Pathogen marginal prevalence in different infection sites. Distribution and spatial trends **(A)** in broader microorganism categories, **(B)** in mixed- and mono-detection cases, **(C)** among primary pathogens, **(D)** among respiratory pathobionts, and **(E)** opportunistic pathogens. Prevalences were analyzed by a logistic model with location modeled as an ordered factor. Red curves depict quadratic trends using intercept, linear and quadratic coefficients from the model. Overall group differences were evaluated using by Wald's test on linear and quadratic coefficients; *p*-values were Bonferroni-adjusted for multiple testing. If the likelihood ratio test was significant, pairwise comparisons of prevalences were performed by Tukey's range test. Significance levels: *p_adj_ < 0.05, ** *p_adj_* < 0.01, ***p_adj_ < 0.001. IFV, influenza virus; PIV, human parainfluenza virus 1/2/3/4; MpV, human metapneumovirus; RSV, human respiratory syncytial virus; AdV, human mastadenovirus of species C, B, or E; BoV, human bocavirus; CoV, human seasonal coronavirus; SARS-CoV-2, severe acute respiratory syndrome coronavirus 2; RhV, human rhinovirus; EntV, non-rhinovirus enteroviruses; mecA, gene for methicillin resistance; URT, upper respiratory tract infections; LRT, lower respiratory tract infections excluding pneumonia; Lung, pneumonia.

The prevalence of primary and opportunistic pathogens was significantly associated with the presumed localization of respiratory infection (Figure 5A, Supplementary Table 4). Both had demonstrated an increasing trend from the upper to the lower respiratory tract ($p_{adj}=\;0.0118$ and $p_{adj}=\;0.02788$, respectively). The trend for primary pathogens, however, was not strictly linear, with significant quadratic effect $\left(\;p_{adj}=0.01756\right)$ indicating that the main rise occurred between URT and LRT, followed by a slight dip from LRT to Lung. *post-hoc* comparisons confirmed higher prevalence in both LRT (ORvs.URT=2.81, $p_{adj}=\;3.71\times 10^{-8}$) and Lung (ORvs.URT=2.27, $p_{adj}\;=\;0.0083$), with insignificant difference between LRT and Lung (OR=1.24, $p_{adj}\;=\;0.7737$). Among opportunists the significant difference had been observed only in Lung vs. URT contrast (OR=1.938, $p_{adj}\;=\;0.0191$). Respiratory pathobionts demonstrated no consistent trend along the tract. Beyond the marginal prevalence of these groups, we analyzed the microbiological composition (exclusively viral, exclusively non-viral, or mixed), but found no overall trend across infection sites (Figure 5B, Supplementary Table 4). Mixed cases, however, showed both a nominal linear  (p=0.0194) and quadratic terms (p=0.0472). Given the prior expectation of mixed infections importance in lower respiratory tract, we additionally performed a likelihood ratio test for this group $\left(\;p=3.18\times 10^{-5}\right)$, and discovered a significant difference between LRT and URT (OR=1.94, $p_{adj}\;=\;0.0001$). We also examined the methicillin resistance gene (mecA) (Figure 5A, rightmost group) and observed its prevalence was greatest in the Lung group (26.52%), but it did not increase robustly toward deeper sites, with its linear term being significant only before correction applied.

Within the primary pathogen group, RSV, IFV, and SARS-CoV-2 were significantly associated with the site of infection (Figure 5C, Supplementary Table 4), each following their typical spatial patterns. RSV demonstrated a sharp rise from URT to LRT, followed by a moderate decline toward Lung, with both linear $\left(\;p_{adj}=8.15\times 10^{-5}\right)$ and quadratic $\left(\;p_{adj}=2.84\times 10^{-8}\right)$ terms being significant; *post-hoc* contrasts supported increased prevalence in lower sites of the tract (LRT vs. URT OR=3.74, $p_{adj}=2.30\times 10^{-14}$, Lung vs. URT OR=2.48, $p_{adj}=\;2.43\times 10^{-5}$), but no significant difference between Lung and LRT was observed. IFV and SARS-CoV-2 each demonstrated a significant quadratic, U-shaped, association with infection site ($p_{adj}=\;0.0324$ and $p_{adj}\;=\;0.0238$ for quadratic effect, respectively). Pairwise comparison partly supports this pattern, demonstrating lower prevalence in LRT than in URT (IFV: OR=0.355, $p_{adj}\;=\;0.0007$, SARS-CoV-2: OR=0.325, $p_{adj}=\;0.0015$), but no difference between LRT and Lung. Among the respiratory pathobionts (Figure 5D, Supplementary Table 4) no omnibus-level significant association between prevalence and location was found. Within the opportunistic pathogens group, *K. pneumoniae* mirrored the overall trend in the joint opportunists group, with significant linear trend $\left(\;p_{adj}=0.01688\right)$ and significantly greater prevalence in Lung as compared to URT (OR=2.122, $p_{adj}\;=\;0.0230$). These associations were robust to adjustment for age group and sampling interval (Supplementary Table 4), with the exception of IFV, whose U-shaped association with infection site was attenuated and did not retain significance, although its lower prevalence in LRT as compared to URT was preserved (adjusted OR=0.355, $p_{adj}\;=\;0.0072$), and also the quadratic component of the primary pathogen trend was likewise attenuated.

### 3.5 Multiple detections

**Figure 6:**
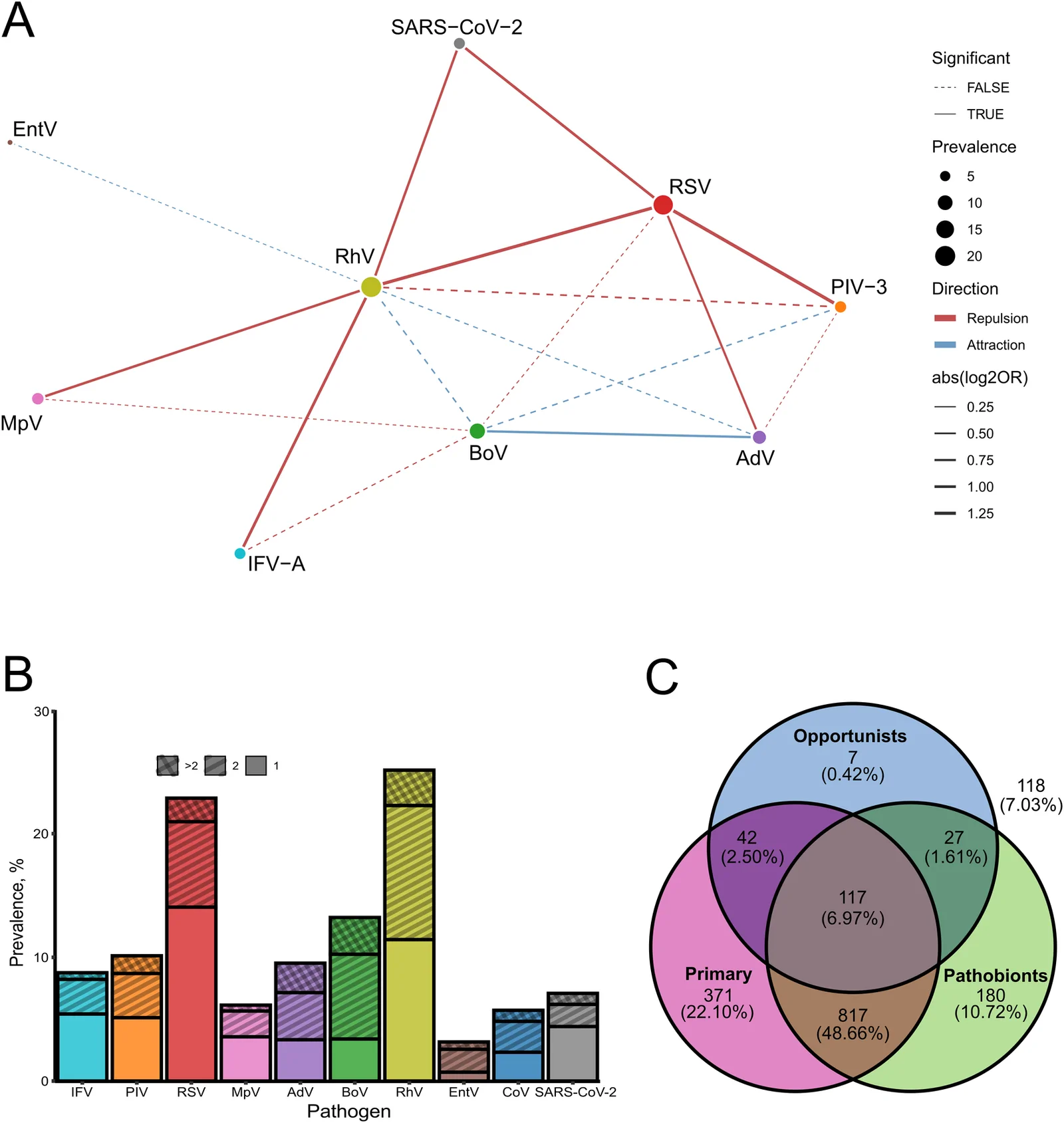
Multiple detections. **(A)** Viral interaction network. Vertices are viruses, for which co-association analysis was performed, their size reflect log10 prevalence. Edges denote interaction (co-association) between viruses, solid lines mark significant interactions — attractions (blue) and repulsions (red). **(B)** Relative proportions of viral detections of varying degree. **(C)** Venn diagram for sets of primary pathogens, opportunistic pathogens, and respiratory pathobionts. IFV, influenza virus; PIV, human parainfluenza virus 1/2/3/4; MpV, human metapneumovirus; RSV, human respiratory syncytial virus; AdV, human mastadenovirus of species C, B, or E; BoV, Human bocavirus; CoV, human seasonal coronavirus; SARS-CoV-2, severe acute respiratory syndrome coronavirus 2; RhV, human rhinovirus; EntV, non-rhinovirus enteroviruses.

Figure 5B hints that most positive cases in the study involved viral-bacterial co-detections. Specifically, only 369 samples (21.98%, 95% CI [20.02, 24.04]) were exclusively viral, while 974 (58.01%, 95% CI [55.61, 60.38]) had a mixed composition, and 216 (12.86%, 95% CI [11.30, 14.56]) contained only bacterial or yeast nucleic acids. The overlaps between different microorganism groups are depicted in the Figure 6C. Even among cases with confirmed viral infection, regardless of concurrent bacterial or fungal detection, instances of co-detection were not rare, accounting for a third of all these cases. The most frequent were cases of dual detection (361 cases, 21.50% overall prevalence, 26.69% of viral cases), triple detections comprised 4.91% of all viral cases, while four- and five-fold detections were observed in twelve and one cases, respectively (Figure 6B, Table 2). BoV and AdV were overrepresented within the latter two groups: BoV appeared in eleven, AdV in eight, and both simultaneously in seven out of thirteen cases. Every virus analyzed participated in co-detections to some degree, though none exclusively (Figure 6A). IFV, MpV, PIV, RSV, and SARS-CoV-2 had the majority of monoinfections, with RSV demonstrating the highest absolute count (236 cases, 61.46% of all RSV detections) and SARS-CoV-2 the highest relative percentage (62.18%, 74 cases). The most promiscuous viruses were picornaviruses RhV and EntV, and BoV: EntV had the highest fraction of co-detections (41 cases, 77.36% of all EntV detections), followed by BoV (165 cases, 74.32%), while RhV yielded the greatest overall co-detection count (230 cases, 54.50%). Of the 17 pairs examined, we identified eight statistically significant associations, with seven pairs (IFV-A-RhV, PIV-3-RSV, MpV-RhV, RSV-AdV, RSV-RhV, RSV-SARS-CoV-2, RhV-SARS-CoV-2) demonstrating repulsion, while the AdV-BoV pair displayed attraction (OR=1.75, $p_{adj}\;=\;0.0447$, see Supplementary Table 5A).

**Table 2:** Viruses co-detected in samples with four or five concurrent viral detections (*n* = 13). Each row represents one sample, columns list the co-detected viruses.

Pathogen 1	Pathogen 2	Pathogen 3	Pathogen 4	Pathogen 5
PIV-3	RSV	AdV	BoV	CoV-NL63
IFV-A	PIV-4	RhV	CoV-NL63	
PIV-3	RSV	BoV	RhV	
RSV	AdV	RhV	SARS-CoV-2	
IFV-A	AdV	BoV	RhV	
RSV	AdV	BoV	RhV	
RSV	BoV	RhV	SARS-CoV-2	
MpV	AdV	BoV	EntV	
AdV	BoV	RhV	EntV	
AdV	BoV	CoV-229E	CoV-NL63	
RSV	BoV	RhV	SARS-CoV-2	
RSV	AdV	BoV	SARS-CoV-2	
RSV	BoV	CoV-OC43	EntV	

IFV-A, influenza virus A; PIV(-3/-4), human parainfluenza virus 3/4; MpV, human metapneumovirus; RSV, human respiratory syncytial virus; AdV, human mastadenovirus of species C, B, or E; BoV, human bocavirus; CoV(-229E/-NL63/OC43), human coronavirus 229E/NL63/OC43; SARS-CoV-2, severe acute respiratory syndrome coronavirus 2; RhV, human rhinovirus; EntV, non-rhinovirus enteroviruses.

The joint group of bacteria and fungi, considered regardless of viral co-detections, was enriched with co-occurrence events, as well: mono-detections accounted for 46.80% (556 cases), dual detection for 31.48% (374 cases), and three- to six-fold co-detections for 21.71% (258 cases). Because most of the microorganisms in the dataset are to some extent common to the host microbiome and only conditionally pathogenic, no co-association analysis was performed within those groups. Instead, we examined pairwise associations between viruses and the microorganisms from these groups. After pruning 74 out of 136 pairs with fewer than ten co-detections, 62 pairs remained for testing. Among these, only two demonstrated significant association: AdV—*H. influenzae* (OR=2.08, $p_{adj}=\;0.003949$) and IFV-A—*M. catarrhalis* (OR=1.92, $p_{adj}\;=\;0.04327$), both were attractions. All ten significant associations, eight virus-virus and two virus-bacterium, retained direction and significance after adjustment for age group and sampling interval in both modeling directions (FDR<0.05 over all 20 directed tests, Supplementary Table 5).

Further testing of the three attraction pairs (AdV-BoV, AdV-*H. influenzae*, and IFV-A-*M. catarrhalis*) for synergistic effects on disease severity, using infection site depth as a proxy for severity, yielded no significant synergy.

### 3.6 Adenovirus fine-typing

**Figure 7:**
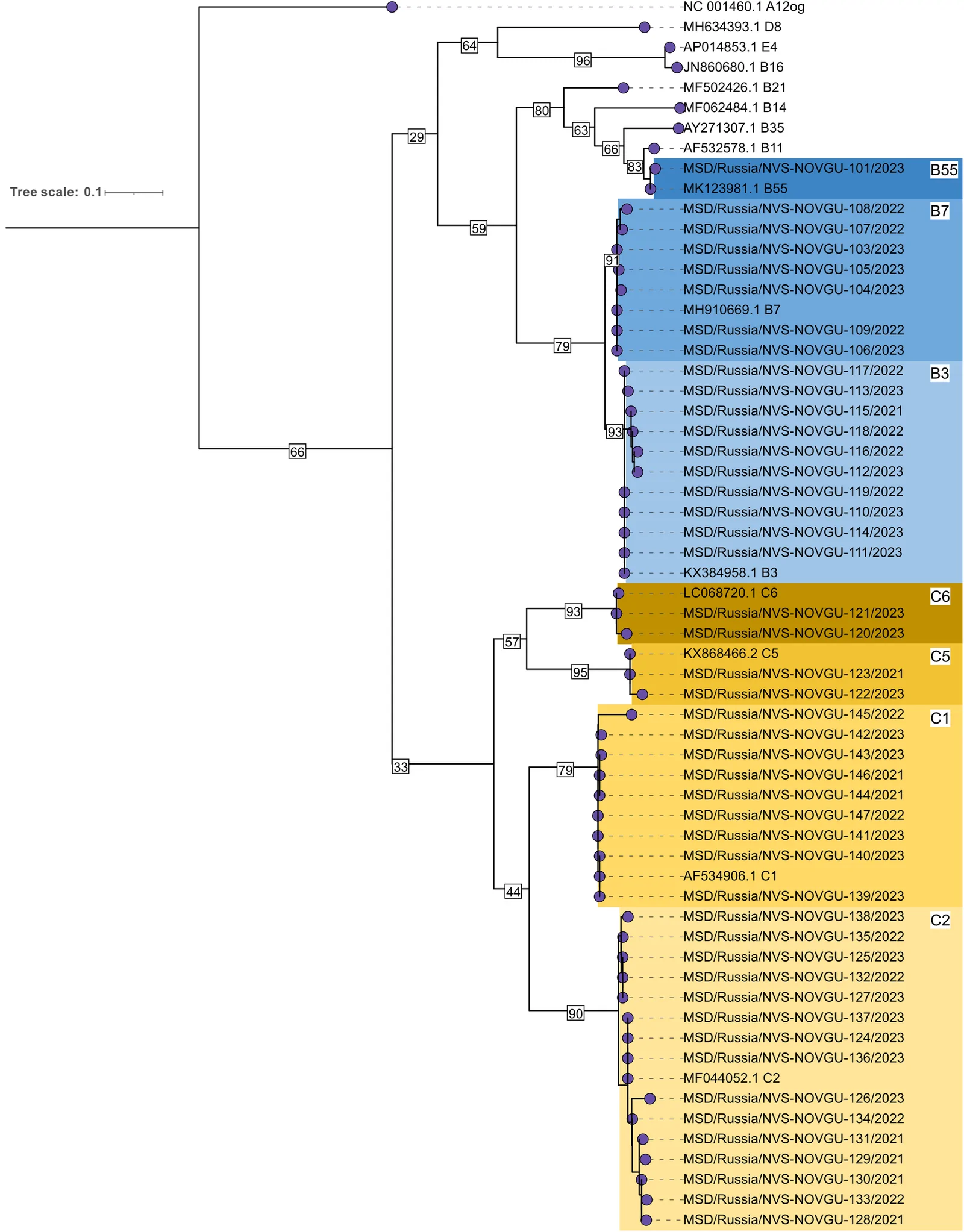
Phylogenetic placement of the fine-typed AdV-positive samples. Clades corresponding to different serotypes are highlighted by different colors. Nodes are labeled by bootstrap support value.

Given that the study period traversed both the height and the decline of the COVID-19 pandemic, when adenovirus-based vaccine Sputnik-V were in wide use, adenoviral genotyping is of particular interest: the predominance or scarcity of vaccine serotypes in clinical isolates could indicate how exogenous introduction of adenovirus may affect the etiological landscape of ARI. Out of 160 AdV-positive samples we selected 50 with Ct ≤ 30 for fine-typing, successfully sequencing 47 samples. Inferred serotypes for 46 samples were supported by both BLAST alignment and phylogenetic analysis. One sequence (MSD/Russia/NVS-NOVGU-102/2023) had lower target region coverage, and therefore was excluded from the tree, though its BLAST hit corresponded to the hexon gene of the AdV-3. Each identified serotype formed a monophyletic clade including its reference sequence, with bootstrap support ranging from 79 to 95 (Figure 7). Thirty-six of the 47 typed samples originated from the E23 interval (Table 3). The most frequent were serotypes AdV-1 and AdV-2 (species C, 9 and 15 cases) and serotypes AdV-3 and AdV-7 (species B, 10 and 8 cases, respectively). Less common were types included AdV-55 (species B, a single case), AdV-5 (species C, vaccine serotype, two cases), and AdV-6 (species C, two cases). Due to sequencing covering only a fragment of the hexon gene, potential recombination events remain invisible.

**Table 3:** Distribution of adenovirus serotypes across sampling intervals among 47 hexon-typed samples.

Serotype	E21	I21	E22	I22	E23	Total
C1	1	0	1	1	6	9
C2	1	0	3	0	11	15
B3	0	1	1	0	8	10
C5	0	1	0	0	1	2
C6	0	0	0	0	2	2
B7	0	0	0	0	8	8
B55	0	0	0	0	1	1

E21, February–April 2021; I21, May–September 2021; E22, October 2021–April 2022; I22, May–September 2022; E23, October 2022–June 2023.

## 4 Discussion

The study analyzed 1,679 pediatric respiratory samples collected in Novosibirsk, Russia, between February 2021 and June 2023, spanning three epidemic and two interepidemic seasons. The main pattern was a high overall positivity rate driven by viral detections (79.15%), with RSV and RhV dominating the etiological structure. The overall viral positivity rate was comparable to the studies performed in similar settings (21, 22). We also assessed oro-nasal presence for a panel of common upper respiratory tract pathobionts; 70.87% of children carried a positive signal for at least one non-viral target. To the best of our knowledge, this represents the broadest diagnostic panel applied to a pediatric respiratory cohort, encompassing both primary pathogens and URT colonizers, providing an unusually comprehensive etiological overview, with only 7.03% of patients being negative for all tested targets.

The study period encompassed both the rise and the decline of the COVID-19 pandemic. SARS-CoV-2 circulation modified the incidence and seasonality of other respiratory viruses (6, 7), and the transition from the peak of the pandemic to its decline is expected to reshape the etiological structure. Across 2021–2023, most viruses retained their characteristic seasonal patterns to some extent.  RSV, IFV, and MpV remained predominantly winter pathogens, while RhV, AdV, and BoV circulated year-round and during interepidemic periods, indicating that the continental pattern of virus seasonality remained generally intact despite the pandemic disruptions. Two notable deviations were observed for MpV and RSV. MpV was nearly absent in the winter-spring 2022, reappearing only in winter 2022–2023 with an atypical early maximum in December. RSV reached its 2021–2022 maximum in October, earlier than usual, and its 2022–2023 peak extended through spring, with a maximum in March. Analogous shifts in seasonality have been reported in a pediatric cohort from Novosibirsk (22) and in a multicenter Russian study encompassing a broader age range (23), with comparable changes described in Switzerland (24) and the USA (25). These findings are further consistent with our systematic review and meta-analysis of RSV circulation across the former Soviet Union (26) and with a recent global meta-analysis of data from 31 countries, which documented widespread alterations in seasonal circulation of multiple respiratory viruses after the onset of the COVID-19 pandemic and a gradual return toward pre-pandemic seasonal patterns (27). Despite the single-center design of the present study, the concordance of our findings with the reports from diverse geographical settings supports the generalizability of the observed post-pandemic seasonal shifts. We interpret these shifts as ecological readjustment after the pandemic peak. This interpretation is further supported by the comparison of the etiological structure across epidemic seasons: the etiological structure was stable between the two later full epidemic seasons (E22 and E23), with only PIV — chiefly PIV-3 — rising and RSV declining from an anomalously large E22 peak to its typical pediatric prevalence. The remaining inter-seasonal variation was driven by the E21 interval which constituted only a trailing half of a full epidemic season (February–April 2021) that inflated the prevalence of spring-peaking viruses (MpV and seasonal coronaviruses) and deflated winter pathogens with early-winter peaks it had missed. Unlike typical seasonal respiratory viruses, the observed differences in SARS-CoV-2 prevalence are unlikely to be attributable solely to the truncation of the sampling interval in E21. The lower SARS-CoV-2 prevalence in E21 and its subsequent increase in E22 coincided with the emergence and rapid spread of the Omicron lineage in Russia (28), which has been associated with increased pediatric hospitalizations compared with earlier SARS-CoV-2 variant (29). Taken together, the relative stability of the etiological structure between E22 and E23 defines a regional baseline against which subsequent surveillance can be evaluated.

Beyond temporal patterns, the dataset highlights structured associations among pathogens. RhV was the most frequently detected virus in our cohort, consistent with the global epidemiology trends (30–32). Although traditionally associated with mild upper respiratory tract infections, RhV is known to cause bronchiolitis in young children (30, 33). Its high clinical significance in ARI, nonetheless, is debated, because of the high detection rates in asymptomatic individuals — up to 80% of positive cases (30, 34). In our data, we found no significant association with infection site, although a slight increase in LRT cases was noted, plausibly reflecting RhV's role as a frequent co-pathogen: 54.50% of RhV-positive patients in our study were co-infections cases. The second most frequent pathogen was RSV, particularly abundant in LRT cases among children under two years of age — the signature distribution of RSV, a leading cause of bronchiolitis in infants (35, 36). BoV, the number three pathogen in our data, had its peak prevalence in children aged 2–6, consistent with the established window for primary infection that extends through early childhood. This high prevalence is intrinsically linked to its tendency for co-detection, which we also observed, with 74.32% BoV-positive cases in our cohort being viral-viral coinfections. Unlike its co-detections with other viruses, which were frequent but showed no significant association, a statistically significant positive association was identified exclusively with AdV. This pair is well-documented, with several explanatory hypotheses proposed: (a) the helper-virus hypothesis, in which AdV directly or indirectly sponsors BoV replication, similar to adeno-associated viruses; (b) the shared tropism hypothesis; and (c) the reactivation hypothesis, based on latent BoV persistence and AdV-induced reactivation (37). In our data, AdV–BoV co-infection showed no association with site of infection, consistent with coexistence in replication dynamics, rather than disease amplification. IFV and SARS-CoV-2, ranked six and eight in the study, both demonstrated a “U”-shaped spatial association. This bimodality is well-documented and reflects clinical severity: early, usually mild infections concentrate in URT during peak shedding; severe pneumonic cases demonstrate prolonged viral detection; and intermediate forms account for the dip in LRT positivity (38, 39). In contrast to pediatric cohorts, both viruses represent major causes of hospitalization among adults (40, 41). For IFV, this is reflected in the linear age-dependent increase in detection rates observed in our cohort, reaching a maximum of 17.68% in the 7–17 year age bracket—a well-recognized pattern attributed to waning maternal antibody protection and increased social exposure (21). SARS-CoV-2 showed a different pattern, with highest positivity among children under two years of age, consistent with epidemiological studies demonstrating elevated infection rates in early childhood (22, 42). While young children show high infection rates, population studies demonstrate that severity increases with age, with elderly patients having the greatest burden of severe onsets and hospitalization, while middle-aged adults experience comparatively lower morbidity (41, 42).

In addition to AdV-BoV pair, the co-detection analysis identified seven viral pairs exhibiting significant negative interaction, that is, pairs in which the presence of one virus was associated with a reduced probability of detecting the other. RSV and RhV occupied central position in the interaction network, each negatively associated with multiple co-circulating viruses. This is a well-aligned pattern, frequently observed in epidemiological studies (8, 43, 44). Notably, RSV and RhV were mutually repulsed, and together with SARS-CoV-2 they formed a repulsion triangle, with each pair having a significant negative association. For the RhV-SARS-CoV-2 edge experimental support exists and is reasonably specific: *in vitro* studies using human airway epithelial cells have shown that RhV inhibits SARS-CoV-2 replication (43, 45), likely through the induction of interferon-stimulated genes that render the epithelium transiently refractory to infection (43, 46). RSV–SARS-CoV-2 interaction is less explored in the literature. One *in vitro* study using nasal human airway epithelial cells reported that prior SARS-CoV-2 infection suppressed subsequent RSV replication, but the reversed does not hold, suggesting asymmetrical interference between two viruses (47). However, experimental evidence for RSV and SARS-CoV-2 interaction remains scarce. And unlike RhV, which maintained its circulation in the COVID-19 pandemic, RSV underwent near-complete suppression during the early pandemic. Combined, these circumstances make distinguishing between biological interference and altered circulation patterns intractable. Of the 249 theoretically possible viral pairs in our panel, we restricted formal interaction testing to the 17 pairs with at least 10 co-detections. For the remaining pairs, low or absent co-detection may hint at repulsion, but given the *a priori* unequal prevalences and different seasonal and age distributions of the viruses in the dataset, such absences cannot be distinguished from the background structure of the data. Within the restricted subset, however, the signal of negative interaction is robust for the pairs highlighted above. These findings extend prior reports of RSV–RhV interference and suggest that similar constraints on co-circulation may apply to other pairings involving these two viruses.

When testing for spatial association across broader pathogen groups, we observed significant trends for the primary pathogens (viruses) and opportunistic pathogenic bacteria, with lower sites showing higher marginal prevalence than in URT. Co-detection stratified analysis did not find any significant spatial trend neither for exclusively viral, nor for exclusively non-viral, nor for mixed cases. Most non-viral targets in our study were common members of the host respiratory microbiome; no evident spatial association was therefore to be expected. This held for all microorganisms, save *K. pneumoniae*, which demonstrated preferential detection in deeper sites of infection. This species is a well-recognized opportunistic pathogen and one of the major causes of nosocomial pneumonia (48), consistent with our observation of its preferential detection in the patients with pneumonia than in those with upper tract infections. Given the recognized importance of viral-bacterial co-infections in respiratory disease, we further examined pairwise viral-bacterial co-detections and identified two statistically significant pairs—IFV-A—*M. catarrhalis* and AdV—*H. Influenzae*. These findings are in line with experimental data, showing that viral infection can sponsor secondary bacterial infections, modulating host immune response and promoting bacterial adherence to host cells (49, 50). However, we detected no associations of these pairs with disease severity using infection site as a proxy. Several limitations should be noted: (a) because all samples were oro-nasal swabs, infection processes in LRT were likely underestimated, particularly for pathogens confined to the bronchi or alveoli; (b) although infection site correlates with ARI severity, it remains an imperfect proxy, influenced by sampling variation and physician subjectivity; (c) empirical antibiotic therapy at the time of hospital admission for respiratory symptoms is a common clinical practice, and the choice of antibiotic class can substantially affect the detection rate of bacterial pathogens. Prior antimicrobial treatment therefore represents an important potential confounder, but this factor could not be accounted for in our analysis due to the lack of systematic data on antibiotic use; (d) members of the pathobiont group may act as primary pathogens by colonizing normally sterile organs and tissues or as opportunistic agents under conditions of impaired host defense. For example, typeable strains of *Haemophilus influenzae*, particularly type b, and specific serotypes of *Streptococcus pneumoniae* are clinically important causes of severe respiratory infections, including pneumonia and meningitis, to the extent that vaccines against them have been developed and incorporated into routine immunization programs (51, 52). At the same time, both species, along with other pathobionts, can be detected in healthy individuals as part of their normal respiratory microbiota, making it difficult to distinguish true infection or opportunistic disease from asymptomatic carriage. Taken together, the co-detection analysis is exploratory rather than confirmatory. Cross-sectional co-occurrence in oro-nasal samples cannot by itself establish biological interaction, as unequal baseline prevalences, shared age and seasonal patterns, and the inability to resolve the direction and timing of co-infection limit causal interpretation. The significant associations are therefore best viewed as prioritized candidate hypotheses; their biological plausibility is discussed above, but their confirmation require further prospective longitudinal and mechanistic studies.

The interpretation of microorganism prevalence is shaped by the nature of PCR-based detection. PCR detects the presence of nucleic acids rather than viable organisms and does so with high sensitivity. A positive signal therefore indicates microbial presence but not necessarily infection (53). While the positive signal for primary pathogens, which are not part of the normal respiratory microbiota, is usually indicative of infection, for the pathobiont group a positive signal may reflect asymptomatic carriage as often as active disease (54, 55), and cannot by itself establish the organism as the cause of infection. This, however, does not make these detections uninformative, as carriage itself is clinically relevant, acting as a reservoir for secondary bacterial infection and a correlate for viral disease severity.

We performed fine-typing of adenoviruses to monitor circulation patterns of specific serotypes, particularly those utilized in vector-based vaccines. AdV was detected in 9.53% of hospitalized children with 47 samples successfully typed using our in-house fine-typing system based on Sanger sequencing of the hexon gene. Most of the samples were collected during the E23 season of the study, characterized by peaking AdV prevalence. AdV-C was the most commonly detected species (60%, 28/47) closely followed by AdV-B (40%, 19/47). The most common serotypes among typed samples were C2, B3 and C1 (in decreasing order). Notably, no AdV-E samples were detected. It is generally accepted that AdV species B and C are the most common species associated with respiratory tract infection worldwide, with serotypes 1–7 accounting for 80% of AdV infection in infants and children (56). However, the most prevalent types vary between different geographic locations over time. AdV-5 is known for its high seroprevalence in the general population reaching the median of 58% in Europe and 71.8% in Asia, although its seroprevalence is reported to be lower in children (57). In our study, only two cases of AdV-5 were detected — one during the 2023 epidemiological season and another in the interepidemic season of 2021. One possible explanation for the low detection of AdV-5 is increased herd immunity following the widespread use of the Ad5/Ad26-based vaccine Sputnik-V during COVID-19 pandemic. However, this hypothesis should be interpreted with caution until further evaluation. We also detected one instance of infection with AdV-55, a serotype associated with severe respiratory illness and considered endemic in China and South Korea. In addition to its sustained circulation in these regions, AdV-55 has been repeatedly implicated in sporadic outbreaks of acute respiratory disease in other parts of the world, highlighting its epidemic potential (58). The first identified AdV-55 variant in Russia was isolated in 2022 (59). More recently, AdV-55 has also been detected among circulating respiratory adenoviruses in Russia in a mixed-aged population study (60). Nevertheless, data on its prevalence among pediatric patients remain scarce, and it has not been previously reported as a commonly circulating type in this population. The modest number of fine-typed samples in our current study does not allow for a robust estimate of AdV serotype prevalence in children with ARI but rather provides preliminary evidence of serotype circulation in the region. It is important to note that molecular surveillance of AdV in Russia is rather limited and these data help address an important regional knowledge gap while highlighting the need for larger longitudinal surveillance studies to better understand AdV circulation patterns.

The study characterized pediatric ARIs in Novosibirsk, Russia across three epidemic and two interepidemic periods (2021–2023) using a broad molecular detection panel. Its analytical breadth allowed for a comprehensive analysis of seasonal and epidemiological patterns, highlighting a trend toward stabilization of the etiological structure to pre-pandemic levels. Incorporation of respiratory tract pathobionts expanded the scope beyond strictly viral cases, and prioritized a number of viral-bacterial associations as clinically relevant hypotheses. Further surveillance will determine whether the trends stabilize, while studies under more controlled settings may help to identify clinically significant pathogen combinations and refine strategies for targeted management of pediatrics ARI. We additionally focused on AdV fine-typing and provided initial evidence for the circulation of specific AdV types among pediatric patients. Although most of the findings support global trends in serotype prevalence, we address a significant knowledge gap in regional AdV epidemiology. Further molecular surveillance of AdV is warranted to detect emerging pathogenic variants.

## Data Availability

The adenovirus sequences generated during the study are deposited in the the VGARus genomic database (accesion numbers novg000053–novg000099) and the GenBank (accesion numbers PZ797416-PZ797462).

## Footnotes

^1^As of June 2023, adenoviral species has been subject to binomial renaming, and now the correct naming for species B, C, and E is *Mastadenovirus blackbeardi*, *M. caesari*, and *M. exoticum* (11). Since the updated naming is not well known among the general scientific community, throughout the paper we use traditional names.

^2^*S. pyogenes* occupies an intermediate position being capable of causing primary pharyngitis, but was grouped among pathobionts due to documented high asymptomatic carriage (8.4–12.9%) (12), its behavior as a transient nasopharyngeal colonizer, and frequent co-detection with other respiratory commensals (4).
